# Conversion surgery intervention versus continued systemic therapy in patients with a response after PD-1/PD-L1 inhibitor-based combination therapy for initially unresectable biliary tract cancer: a retrospective cohort study

**DOI:** 10.1097/JS9.0000000000001540

**Published:** 2024-05-03

**Authors:** Shanshan Wang, Yunchao Wang, Chengpei Zhu, Kai Liu, Jiashuo Chao, Nan Zhang, Mingjian Piao, Xu Yang, Longhao Zhang, Junyu Long, Ziyu Xun, Ting Zhang, Xinting Sang, Xiaobo Yang, Haitao Zhao

**Affiliations:** a Department of Liver Surgery, Peking Union Medical College Hospital, Chinese Academy of Medical Sciences and Peking Union Medical College (CAMS and PUMC); b Department of General Surgery Center, Beijing Youan Hospital, Clinical Center for Liver Cancer, Capital Medical University, Beijing; c Organ Transplantation Center, The First Affiliated Hospital of Shandong First Medical University, Jinan; d Department of General Surgery, Digestive Disease Hospital Affiliated to Zunyi Medical University, Affiliated Hospital of Zunyi Medical University Zunyi, Guizhou, People’s Republic of China

**Keywords:** chemotherapy, conversion surgery, immune checkpoint inhibitors, PD-1/PD-L1 inhibitors, unresectable biliary tract cancer

## Abstract

**Background:**

The role of conversion surgery in patients with unresectable biliary tract cancer who responded positively to PD-1/PD-L1 inhibitor-based therapy remains unclear. This study aimed to assess the outcomes in patients with or without conversion surgery.

**Methods:**

In this cohort study, patients with advanced biliary tract cancer who received combination therapy with PD-1/PD-L1 inhibitors from July 2019 to January 2023 were retrospectively. Patients who exhibited positive responses and met the criteria for conversion surgery were enrolled, and their surgical and oncological outcomes were analyzed.

**Results:**

Out of 475 patients, 34 who met the conversion resection criteria were enrolled. The median follow-up was 40.5 months postinitiation of systemic therapy. Ultimately, 13 patients underwent conversion surgery, while 21 received continuation of systemic treatment alone (nonsurgical group). The median interval from the initial antitumor therapy to surgery was 6.7 [interquartile range (IQR) 4.9–9.2] months. Survival with conversion surgery was ﻿significantly longer than the nonsurgical cohort, with a median progression-free survival (PFS) [unreached vs. 12.4 months; hazard ratio 0.17 (95% CI: 0.06–0.48); *P*=0.001] and overall survival (OS) [unreached vs. 22.4 months; hazard ratio 0.28 (95% CI: 0.09–0.84); *P*=0.02], respectively. ﻿After a median postoperative follow-up of 32.2 months in the surgical cohort, eight patients survived without recurrence. The estimated 3-year OS, PFS, and recurrence-free survival rate in the surgical cohort were 59.9, 59.2, and 60.6%, respectively. The R0 resection rate reached 92.3%, with two achieving a pathological complete response. One patient experienced a Clavien–Dindo grade 3 complication without surgery-related mortality. No serious adverse events or surgical delays were observed. Multivariate analysis indicated that conversion surgery was independently associated with OS (*P*=0.03) and PFS survival (*P*=0.003).

**Conclusion:**

Conversion surgery appears safe and offers survival benefits to patients responding to immune checkpoint inhibitors-based combinations. However, further studies are required to validate this strategy in the era of immunotherapy.

## Introduction

Highlights

- Conversion surgery after upfront immune checkpoint inhibitors (ICIs)-based combinations for unresectable biliary tract cancers is feasible with satisfactory safety profiles.
- ﻿The addition of subsequent conversion resection following good response to ICIs-based combinations confers additional survival benefits compared to those without surgery.
- Surgery and systemic therapy could become complementary within the whole-process management of advanced biliary tract cancers in the immunotherapy era, pursue surgical resection possibilities during ICIs-based therapy should be proactively carried out.

Surgical resection stands as the sole curative treatment for biliary tract cancers (BTCs). Regrettably, only around 35% of patients qualify for curative resection due to advanced-stage diagnosis^1^. Standard chemotherapy for advanced BTC typically results in limited survival, ~1-year^2^. The addition of immune checkpoint inhibitors (ICIs) to chemotherapy may extend survival by about a month, rarely surpassing 2 years^3,4^.

Growing evidence suggests that downstaging followed by conversion surgery subsequent to initial systemic or local treatment offers opportunities for radical resection, reduced recurrence, and improved survival in other tumors^5–7^. However, data on conversion surgery in BTC remains scarce due to the limited efficacy of chemotherapy. Case reports or small case series evaluating neoadjuvant or conversion chemotherapy have shown prolonged survival in patients with objective response^8–11^.

The emergence of ICIs has transformed the therapeutic landscape for initially unresectable BTC. Combining chemotherapy, tyrosine kinase inhibitors (TKIs), or local treatment can enhance the efficacy of immunotherapy^12,13^. The role of conversion surgery may evolve with modern treatment regimens in the ICI era. ICIs-based combinations have demonstrated promising response rates ranging from 26.7 to 80% for BTC^3,14,15^. Consequently, tumor downsizing, or even downstaging, has become more common in practice, and this unprecedented high antitumor efficacy has made conversion therapy possible for suitable candidates. The potential of this strategy is to facilitate surgical removal of the tumor in unresectable BTC cases. As previously demonstrated, this combination therapy yielded an objective response rate (ORR) of 32%, with three patients undergoing surgery with the combined therapy^12,16^. However, data on the role of additional conversion resection in patients with initially unresectable BTC who respond favorably to systemic immunotherapy^12,16,17^, along with findings from the chemotherapy era, may not be directly applicable in the current era of ICIs due to differing mechanisms of action^18^.

Here, we explored the oncological outcomes of the addition of conversion surgery and continuation of systemic treatment alone in patients who responded well to ICIs-based therapy, alongside with the surgical outcomes of conversion surgery.

## Materials and methods

### Study design and patients

Between July 2019 and January 2023, we retrospectively screened consecutive patients with histologically confirmed advanced BTC ﻿who received at least two cycles of combination therapy based on PD-1/PD-L1 inhibitors at Peking Union Medical College Hospital (PUMCH). Combination therapy refers to the use of PD-1/PD-L1 inhibitors alongside targeted agents, chemotherapy, or local therapy. Unresectability was assessed by a multidisciplinary team (MDT) and was defined as the inability to achieve an R0 resection, even with proactive surgical procedures such as combined vascular resection, presence of distant metastases, or inability to tolerate curative liver resection (e.g. due to insufficient remaining liver volume). Patients who met the criteria for conversion surgery, as outlined in the conversion surgery section, after ICIs-based combination therapy were included. Exclusion criteria for this cohort study were as follows: (1) BTC remnants and recurrence and (2) the presence of other malignancies.

This study has been reported in line with the strengthening the reporting of cohort, cross-sectional, and case–control studies in surgery (STROCSS) criteria^19^ (Supplemental Digital Content 1, http://links.lww.com/JS9/C468) and performed in accordance with the Declaration of Helsinki and approved by the Ethics Committee of PUMCH (No.JS-1391). It has been registered at ClinicalTrials.gov (NCT03892577 ).

### Treatment and data collection

PD-1/L1 inhibitors were administered every 3 weeks at varying doses: pembrolizumab^20^, camrelizumab^21^, sintilimab^22^, and tislelizumab^17^ (200 mg), toripalimab^23^ (240 mg), or devalumab^3^ (1500 mg), or envafolimab^24^ (2.5 mg/Kg). Lenvatinib was orally administered given orally once daily (8 mg/day for body weight <60 kg or 12 mg/day for body weight ≥60 kg)^25^. The primary chemotherapeutic regimen consisted of gemcitabine plus oxaliplatin (Gemox)^25^. The choice of systemic regimen was determined based on patient preference following a comprehensive discussion of the latest efficacy and safety data, treatment cycles, and cost. The decision to proceed with local therapy (e.g. radiotherapy) depended on the MDT’s opinion.

Information regarding treatment initiation and completion dates, initial doses, radiological evaluations, laboratory data, surgery data, and adverse events (AEs) during treatment were systematically collected.

### Assessments and endpoints

Tumor assessments were conducted every 6–9 weeks using computed tomography (CT) or MRI scans, evaluated by experienced radiologists using RECIST v1.1. Positron emission tomography (PET) scans were also used selectively. AEs were assessed using the National Cancer Institute Common Terminology Criteria for Adverse Events (CTCAE) version 5.0.

Study endpoints comprised progression-free survival (PFS; the time interval from ICIs initiation to progression, ﻿recurrence, last follow-up, or death), recurrence-free survival (RFS; the time interval from surgery to first recurrence, progression, last follow-up, or death), overall survival (OS; the time interval from ICIs initiation to death or last follow-up), safety, postoperative hospital stay, and surgical complications classified as described by Clavien *et al*.^26^ The time from ICIs administration to surgery was also analyzed.

### Indication and procedure of conversion surgery

Candidates for conversion surgery should demonstrate a confirmed positive response as partial response (PR) or shrinkage-stable disease (SD) assessed by CT or MRI of the chest, abdomen, and pelvis and PET-CT was requested. Indications for surgery included (1) hepatic lesions showing a radiographic good response for ≥1 month; (2) absence of distant metastases or metabolic activity on PET-CT for ≥1 month; (3) stabilization of tumor markers; (4) technically resectable vascular tumor thrombus; (5) preservation of sufficient remnant liver volume; (6) absence of contraindications for hepatectomy. R0 resectability was assessed by an experienced MDT.

Subsequent resection was performed after consultation, obtaining informed consent. It is essential to adequately explain the risks and benefits of surgery. The decision to proceed with conversion surgery depends on the patient’s autonomy. The resection approach was determined on a case-by-case basis. A laparoscopic, laparotomy, or conversion-to-laparotomy approach was adopted based on the extent and complexity of the case. Suspicious metastatic lesions were excluded from frozen section evaluation. A single experienced surgical team performed all surgeries.

Before conversion surgery, TKIs and ICIs were discontinued for at least 1 week. A pathological complete response (pCR) was defined as the absence of residual viable tumor cells with hematoxylin and eosin staining on slide sections from completely resected primary tumors or metastatic lesions.

### Postoperative management and follow-up

Presurgical systemic therapy was resumed once the patients were fully recovered from surgery (approximately 3–4 postoperative weeks). The choice and duration of postoperative systemic therapy was made through the MDT based on the original response, postoperative clinical conditions, and patient’s willingness. When tumor recurrence is diagnosed, the subsequent treatment is based on the pattern of tumor recurrence, clinical conditions, and other factors. Nonsurgical patients were advised to continue the initial effective therapy.

Enhanced MRI/CT scans were performed every 2–3 months or when recurrence was suspected based on elevated serum tumor biomarkers. PET-CT was conducted if clinically indicated. The endpoint of follow-up was 5 January 2024.

### Statistical analysis

Continuous variables are presented as median, range, or interquartile range (IQR), and compared using the unpaired *t*-test or the Mann–Whitney *U* test where appropriate. Categorical variables are shown as frequencies and percentages and were compared using Fisher’s exact test. Survival analyses were conducted using the Kaplan–Meier method and compared using the log-rank test. A Cox regression model was applied to perform multivariate analysis. Statistical significance was set at *P*<0.05. Statistical analyses were performed using the SPSS version 27.

## Results

### Patient characteristics

**Figure 1:**
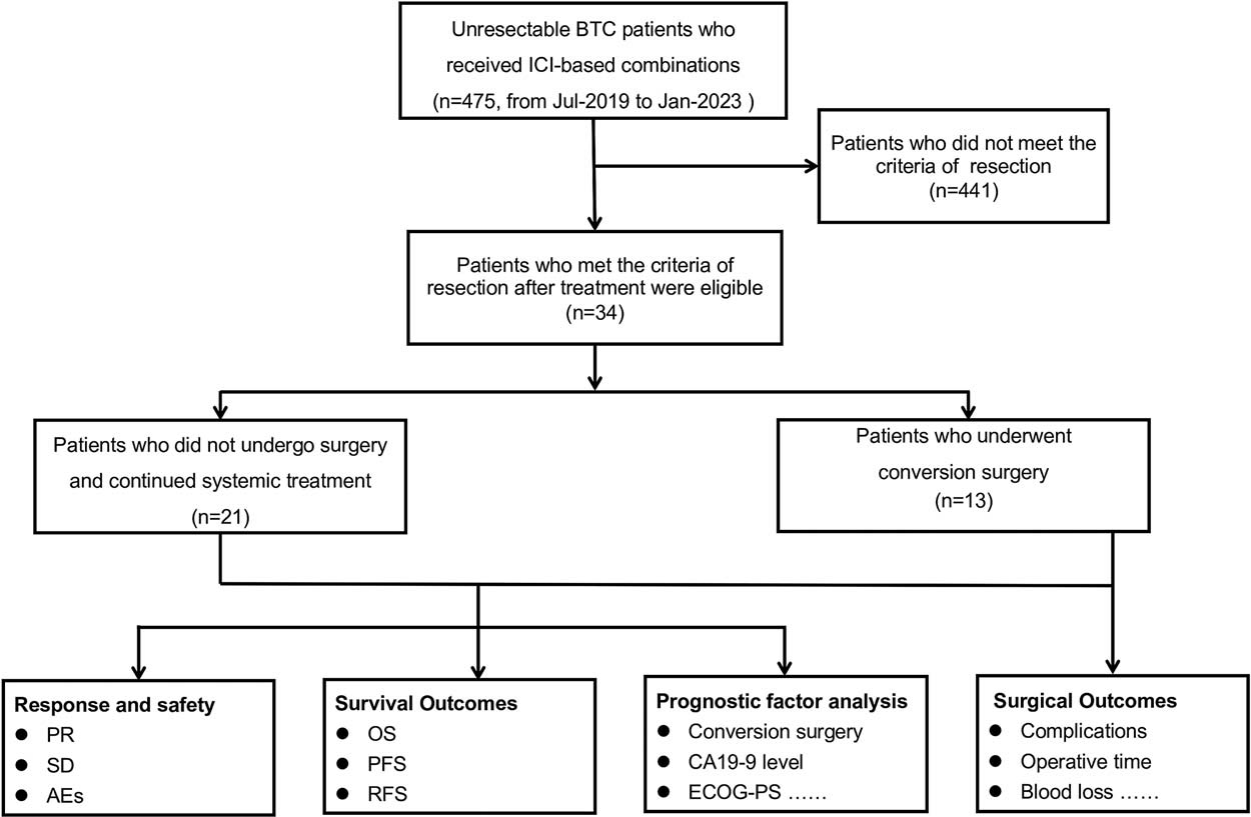
Flowchart. AEs, adverse events; BTC, biliary tract cancer; CA 19-9, carbohydrate antigen 19-9; ECOG-PS, Eastern Cooperative Oncology Group Performance Status; OS, overall survival; PFS, progression-free survival; PR, partial response; RFS, recurrence-free survival; SD stable disease.

Between July 2019 and January 2023, 475 patients underwent screening, of which 34 met the eligibility criteria (Fig. 1). Among the 34 cases, 13 (38.2%) underwent conversion surgery, while 21 (61.8%) declined surgery due to perceived the surgical risks and uncertain benefits. Baseline demographics and disease characteristics were generally balanced between the two groups (Table 1). The median age of the entire cohort was 61years (range 40–77), with 38.2% (13/34) being female, 61.8% (21/34) had an Eastern Cooperative Oncology Group performance status (ECOG PS) of 0 and 88.2% (30/34) were categorized as Child-Pugh class A. Among the 34 patients, 29 (85.3%) had metastatic disease, 20 (58.8%) had ﻿tumors of intrahepatic origin, and 14 (41.2%) had gallbladder cancer (GBC). The most common of metastatic sites were the lymph node metastases (79.4%). Additionally, three patients underwent conversion surgery after second-line treatment.

**Table 1:** Baseline characteristics.

	No. (%)			
Characteristic	Total (*N*=34)	Surgical group (*n*=13)	Nonsurgical group (*n*=21)	*P*
﻿Median age (range) — year	61 (40–77)	62 (41–77)	58 (40–72)	0.15
﻿Female	13 (38.2)	6 (46.2)	7 (33.3)	0.49
ECOG PS﻿				
0	21 (61.8)	9 (69.2)	12 (57.1)	0.72
1	13 (38.2)	4 (30.8)	9 (42.9)	
Child-Pugh grade﻿				
A	30 (88.2)	13 (100)	17 (81.0)	0.14
B	4 (11.8)	–	4 (19.0)	
Extent of disease﻿				
Locally advanced	5 (14.7)	3 (23.1)	2 (9.5)	0.35
Metastatic	29 (85.3)	10 (76.9)	19 (90.5)	
﻿Primary tumor site﻿				
GBC	14 (41.2)	7 (53.8)	7 (33.3)	0.30
ICC	20 (58.8)	6 (46.2)	14 (66.7)	
﻿Metastatic site﻿				
Lymph nodes	27 (79.4)	11 (84.6)	16 (76.2)	0.68
Liver	24 (70.6)	9 (69.2)	15 (71.4)	1.0
Lung	4 (11.8)	2 (15.4)	2 (9.5)	0.63
Bone	6 (17.6)	1 (7.7)	5 (23.8)	0.37
Peritoneum	9 (26.5)	5 (38.5)	4 (19.0)	0.25
﻿Baseline CA19-9 (U/ml) (median, IQR)	39.2 (14.8–477.8)	35.2 (15.1–276.6)	52.0 (14.4–587.4)	0.71
Treatment line﻿				
1	22 (64.7)	10 (76.9)	12 (57.1)	0.29
2	12 (35.3)	3 (23.1)	9 (42.9)	

CA19-9, carbohydrate antigen 19-9; ECOG PS, Eastern Cooperative Oncology Group Performance Status; GBC, gallbladder cancer; ICC, intrahepatic cholangiocarcinoma; IQR, interquartile range.

### Tumor Response, safety, and oncological outcomes

**Figure 2:**
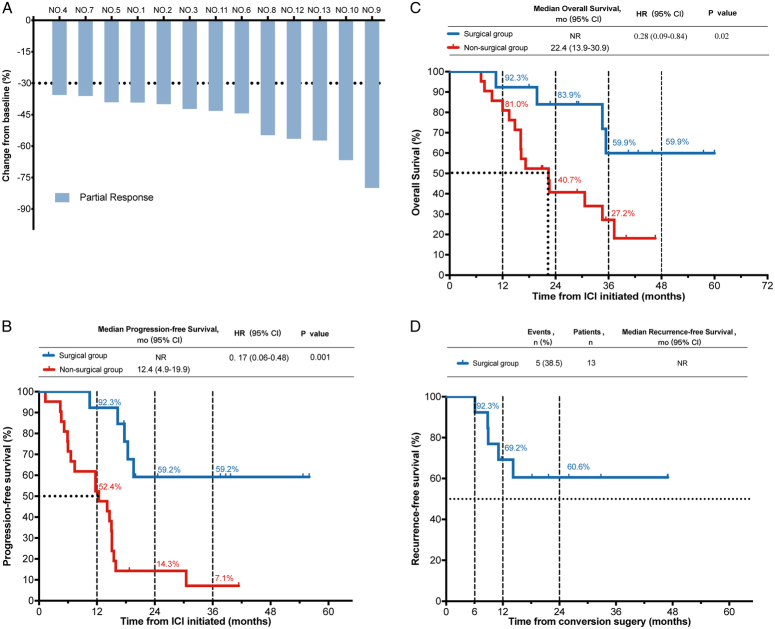
Efficacy outcomes. (A) Response for target lesions before resection in surgical group; **﻿**Kaplan–Meier Curves in the surgical group and nonsurgical group: progression-free survival (B), overall survival (C), Recurrence-free survival of surgical group (D). ICI, immune checkpoint inhibitor.

All 34 patients who met the criteria for resectability achieved PR (Fig. 2A; Table 2). Demographics and disease characteristics at the time of meeting the conversion surgery criteria (Supplementary Table S1, Supplemental Digital Content 2, http://links.lww.com/JS9/C469) were generally balanced between the two groups. As of the data cutoff on 5 January 2024, the median follow-up time was 40.5 ﻿(range 7.1–60.0) months. In the nonsurgical group, the median PFS and OS was 12.4 (95% CI: 4.9–19.9) months and 22.4 (95% CI: 13.9–30.9) months, respectively. In the surgical group, nine (64.3%) were alive, with eight (61.5%) surviving without tumor recurrence. The median PFS (1-year, 2-year, 3-year survivals, 92.3%, 59.2%, 59.2%, respectively), OS (1-year, 2-year, 3-year survivals, 92.3%, 83.9%, 59.9%, respectively), and RFS (1-year, 2-year, 3-year survivals, 69.2%, 60.6%, 60.6%, respectively) were not reached (Fig. 2B, C, D). Compared to the nonsurgical group, patients who underwent resection had significantly longer PFS (HR=0.17, 95% CI: 0.06–0.48; *P*=0.001) and OS (HR=0.28, 95% CI: 0.09–0.84; *P*=0.02) (Fig. 2B, C; Table 2). Multivariable Cox regression analysis performed on the entire cohort of 34 patients identified only conversion surgery as an independent factor positively associated with PFS (HR=0.20, 95% CI: 0.06–0.55; *P*=0.003) and OS (HR=0.29, 95% CI: 0.05–0.29; *P*=0.03), respectively (Supplemental Table S2, Supplemental Digital Content 3, http://links.lww.com/JS9/C470).

**Table 2:** Therapeutic efficacy, surgical, and oncological outcomes of two groups.

Parameter	Surgical group (*n*=13)	Nonsurgical group (*n*=21)	HR (95% CI) *P*
Partial response (PR), *n*, (%)	13 (100)	21 (100)	
Objective response rate (ORR), *n*, (%)	13 (100)	21 (100)	
Progression-free survival (PFS), months, median, (95% CI)	NR	12.4 (4.9–19.9)	0.17 (0.06–0.48) *P*=0.001
PFS rate (%)			
1-year	92.3	52.4	
3-year	59.2	7.1	
Recurrence-free survival rate (RFS) (%)			
1-year	69.2	–	
3-year	60.6		
Overall survival (OS) months, median, (95% CI)	NR	22.4 (13.9–30.9)	0.28 (0.09–0.84) *P*=0.02
OS rate (%)			
1-year	92.3	81.0	
3-year	59.9	27.2	
Interval from initial ICI to surgery months, median, (IQR)	6.7 (4.9–9.2)	–	
Interval from TKI discontinued to surgery, days, median, (IQR)	11.0 (9.0–15.0)	–	
Interval from ICI discontinued to surgery, days, median, (IQR)	23.0 (21.0–28.5)	–	
Surgical approaches			
Laparoscopic	6	–	
Laparotomy	7^a^		
Operation time, min, median, (IQR)	220.0 (180.0–242.5)	–	
Length of postoperative hospital stay, days, median, (IQR)	8 (6–10)	–	
Blood loss, ml, median (IQR)	200 (100–200)	–	
Blood transfusions, *n*, (%)	1 (7.7)	–	
Postoperative complications≥Clavien–Dindo^b^ 3a﻿	1 (7.7)	–	
90-Day mortality, *n*, (%)	0	–	
R0 resection, *n*, (%)	12 (92.3)	–	
Pathologic complete response (pCR), *n*, (%)	2 (15.4)	–	

a One patient underwent laparoscopic exploration and then was converted to laparotomy.

b Clavien–Dindo classification of surgical complications.

HR, hazard ratio; IQR, interquartile range; NR, not reach.

### Therapeutic regimens, perioperative conditions, and outcome in the surgical group

**Figure 3:**
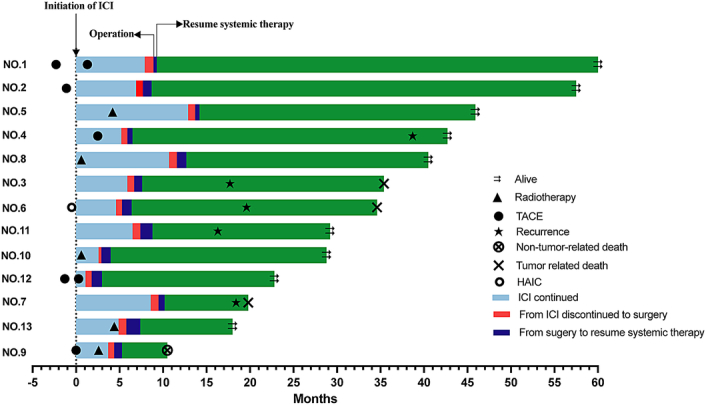
Swimming chart showing duration of treatment in surgical group. ICI, immune checkpoint inhibitor; HAIC, hepatic artery infusion chemotherapy; TACE, transarterial chemoembolization.

Details of the 13 patients who underwent conversion surgery are presented in Figure 3 and Supplemental Table S3 (Supplemental Digital Content 4, http://links.lww.com/JS9/C471). Among them, six ﻿patients had intrahepatic cholangiocarcinoma (ICC) and seven had GBC. Eleven (84.6%) patients had metastatic lesions with distant lymph nodes being the most frequent site (61.5%). Additionally, five (38.5%) had peritoneal carcinomatosis, nine (69.2%) had intrahepatic metastasis, and two (15.4%) had lung metastasis. Ten (76.9%) patients received first-line systemic therapy and three patients received second-line therapy. Six patients were treated with PD-1 inhibitor and TKI agents plus local-regional therapies, while two received PD-L1 combined chemotherapy. Five patients with metastases underwent radiotherapy. Transarterial chemoembolization (TACE) was delivered to in five patients and one accepted hepatic artery infusion chemotherapy (HAIC). The median interval from the initial ICIs to surgery was 6.7 (IQR 4.9–9.2) months. Six patients underwent laparoscopy. The average blood loss was 200 ml (range 30–200), and the median postoperative hospital stay was 8 (IQR 6–10) days. Generally, surgical resection was safe, with only one patient experiencing a Clavien–Dindo grade 3 complication, and all patients were discharged without surgery-related mortality. Pathological analysis showed an R0 resection rate of 92.3% (12/13), with two patients (15.4%) achieving pCR (Table 2). One patient (NO.2) underwent resection of an abdominal wall lesion but was confirmed to have residual cancer cells. As of the data cutoff, the median postoperative follow-up time was 32.2 (IQR 16.6–34.5) months, and four patients experienced tumor recurrence, with recurrence sites including the liver, colon, and hilar lymph node. Notably, patient No 2, with R1 resection, did not exhibit recurrence and remained alive and in good condition.

All 13 patients fully recovered from surgery, and upfront antitumor systemic therapy was resumed. Throughout the entire cohort, all patients experienced AEs of various grades during antitumor therapy; however, no surgical delays due to AEs or grade 4–5 AEs occurred.

## Discussion

﻿﻿In the era of ICIs, to our knowledge, this study represents the first and largest analysis of short-term and long-term outcomes of conversion surgery following combination therapy with PD-1/PD-L1 inhibitors for unresectable BTC. Our results suggest that initially unresectable BTC can be converted via immunotherapy-based combinations with satisfactory safety profiles, and the addition of conversion resection confers additional survival benefits compared to those without resection, even in cases of metastatic BTC. These findings offer valuable evidence for integrating conversion resection into future BTC treatment algorithms.

Currently, controversy persists regarding the feasibility and validity of conversion surgery for BTC, prompting substantial clinical debate. One key issue is the variability in the definition of ‘initially unresectable’ among different institutions and practitioners, with most studies evaluating conversion surgery including patients with locally advanced disease^27^. However, there is a significant distinction concerning ‘unresectable due to locally advanced’ cases. Reported incidence rates of conversion vary widely from 0.65 to 80%^27,28^, and the R0 resection rate ranged from 4 to 83%^27,28^. The postulated reason for the substantially variable outcomes might be due to the uniform unresectable criteria and that many patients had received neoadjuvant treatment^27,29^, making it challenging to accurately assess the efficacy of conversion surgeries. To more accurately evaluate the clinical impact of conversion surgery, we excluded patients initially considered technically resectable and those who received neoadjuvant therapy, enrolling only obviously unresectable patients. Our findings indicate that conversion surgery via combined therapy based on PD-1/PD-L1 inhibitors is feasible in a proportion of such patients. However, regarding candidates for conversion therapy, our results could not present any solid data due to the study design.

The improved response rates achieved with systemic therapies have raised questions about whether patients might benefit more from conversion surgery rather than systemic therapy alone. In 2015, Kato *et al*.^11^ reported that resection following systemic chemotherapy resulted in a significantly longer survival, yielding a 2-year OS rate of 45.0% compared with 19.0% in patients who were unable to undergo surgery. Another retrospective, multicenter analysis in 2020 included 24 patients undergoing conversion surgery demonstrating a 5-year OS rate of 43.2% with a mean OS of 57.6 months, which was significantly better than that for chemotherapy only (*P*<0.001)^27^, which is consistent with our findings. Notably, previous studies compared patients whose lesions achieved resectable criteria with those whose disease remained unresectable, leading to biased comparison, as patients undergoing conversion surgery may response better to chemotherapy, and even without resection, they may still survive longer than those in the nonsurgical group. In our study, patients in both surgical and nonsurgical groups met resectability criteria following treatment, allowing for a fair comparison of outcomes. We demonstrated that PFS and OS were significantly better in the surgical group. These results may be explained by the limitations of systemic therapy, as most patients develop resistance after initial remission. The median PFS of first-line treatment with ﻿Pembrolizumab/Durvalumab plus Gemcitabine and Cisplatin (GC) is 6.5–7.2 months, with only 0.6–2.1% achieving complete response (CR) from systemic therapy alone^3,4^. The shortest duration of response (DOR) observed was 4.6 months^3^, suggesting the possibility of progression after achieving ORR 6 months later. Moreover, second-line regimens are lacking^1^. Therefore, until the ﻿systemic therapies undergo radical evolution, proactive resection may prove beneficial for selected patients in whom lesion shrinkage allows for surgery.

The third issue revolves around establishing potent conversion therapy for unresectable BTC. Conversion surgery requires a highly objective remission to create surgical opportunities, and the ORR can be used as a guiding criterion. The response rate to chemotherapy alone has been unsatisfactory (<20%)^2^. Recently, immunotherapy-based combination therapies have garnered increasing interest. ICIs plus chemotherapy have shown a higher ORR compared to chemotherapy alone (29.0 vs. 18.7%)^4^. Moreover, growing evidence indicates that inhibiting angiogenesis by targeting VEGF/VEGFR can enhance the antitumor effects of ICIs^18,30^. Emerging data have shown the value of adding locoregional therapies (e.g. radiotherapy) with highly effective targeted agents and ICIs in downstaging or downsizing hepatobiliary tumors^6,15,21^. The therapeutic benefits of each modality can be enhanced to produce a synergistic effect^31^. Many studies have demonstrated the efficacy and safety of ICIs plus lenvatinib, with an ORR of 25–42.1%^15,20^. In 2022, a phase 2 study involving 38 patients with unresectable BTC showed that ICIs plus lenvatinib as first-line treatment resulted in an ORR of 42.1%, and 34.2% achieved downstaging and underwent surgery, with a major pathologic response (MPR) of 46.2%^15^. The event-free survival was extended to 13.5 months. Results from a prospective clinical trial including 30 advanced ICC patients treated with toripalimab combined with lenvatinib and GEMOX demonstrated an ORR of 80% with a median OS, PFS, and DoR of 22.5, 10.2, and 11.0 months, respectively^14^. Additionally, a recent a real-world study to assessed this regimen as a beyond first-line therapy for advanced BTC, revealing a median PFS of 9.3 months, a median OS of 13.4 months and an ORR of 43.9% with tolerable toxicity^25^. Furthermore, our center observed that adding local treatment early in the treatment course, based on TKI and ICI, potentially enhanced tumor response and extended survival^12^. While this combination represents a potential option for conversion treatment in well-selected patients, high-level evidence from well-designed trials is still warranted.

The optimal timing of conversion surgery remains a contentious topic of debate in whose tumor becomes resectable after anti-cancer therapy, for other cancers either^18,27^. Kato *et al*.^11^ showed that the mean duration of chemotherapy required for downsizing for surgical resection was ~6 months. Some studies recommend re-evaluation of the response when the tumor is resectable to observe its biological behavior and determine whether to perform surgery. Creasy *et al*.^9^ reported the median time from response assessment to the surgery was 51 days. Overall, the timing of systemic therapy and conversion surgery remains a nuanced decision with no singular approach, varying from 3 to 6 months^11,28,32^. At our center, the timing of surgery is determined through a comprehensive evaluation of the initially unresectable causes, speed and depth of regression, and AEs. The crux of timing selection revolves around the meticulous evaluation of tumor response, involving radiological and serological assessments, along with dynamic observations to ascertain the stability and sustainability of the therapeutic response. Multidimensional assessment (CT, MRI, and PET-CT) may be necessary (detailed in ‘Indication and procedure of surgery’ subsection). Based on these preliminary findings, aggressive surgical resection should be considered for patients with sustained favorable responses to ICI-based therapy. We view resection as a distinct form of ‘local-therapy’ within the whole-process management of advanced BTC. The long-lasting effects of ICIs may encourage the complete removal of residual masses, as evident by a patient with R1 resection who remains under active surveillance with no evidence of disease progression 34 months postoperatively. One plausible biological explanation for the survival benefit of R1 resection could be the negative immunomodulatory effects of tumor cells^18^. Notably, a timely resumption of systemic therapy appears pivotal for managing these patients. Overall, the decision to recommend conversion surgery must be personalized, relying on the clinical judgment of an experienced MDT, considering both operation-related, host-related, and tumor-related factors.

This study had some limitations. Firstly, due to its retrospective nature and small sample size, the generalizability of the findings is limited. However, given the evolving landscape of immunotherapy for advanced BTC, initial exploration of the feasibility, effectiveness, and safety of conversion surgery is crucial. Owing to the rarity and heterogeneity of BTC, multicenter or worldwide prospective studies are needed to confirm these findings. Secondly, criteria for resectability and surgical approaches remain insufficient, as a technical appreciation of the feasibility of tumor removal relies heavily on the experience of surgical teams. Nonetheless, conversion surgery meeting these criteria appears to be effective based on our results. Lastly, considerable inconsistencies exist in various aspects, including regimens, surgical approaches, and timing of surgery, necessitating further elucidation. These inconsistencies stem from the complexity of real-world situations, such as individualized differences, insurance coverage, and patient preferences. Treatment decisions were made with patient consent and in compliance with ethical guidelines and the compassionate use principle.

## Conclusion

Conversion surgery for biliary malignancies may be feasible after combinations with PD-1/PD-L1 inhibitors. Subsequent resection following a good response could potentially offer significant survival benefits ﻿compared to those who do not undergo surgery. The role of this strategy in the immunotherapy era requires further formal investigation.

## Ethical approval

The study was conducted in accordance with the Declaration of Helsinki, and approved by the Institutional Review Board (IRB) and Ethics Committee (EC) of PUMCH (No. JS-1391).

## Consent

Written informed consent was obtained from the patient for publication and any accompanying images. A copy of the written consent is available for review by the Editor-in-Chief of this journal on request.

## Sources of funding

This research was funded by National High-Level Hospital Clinical Research Funding [2022-PUMCH-B-128], CAMS Innovation Fund for Medical Sciences (CIFMS) ([2022-I2M-C&T-A-003], [2021-I2M-1-061], [2021-I2M-1-003]), CSCO-hengrui Cancer Research Fund ([Y-HR2019-0239], [Y-HR2020MS-0415], [Y-HR2020QN-0414]), CSCO-MSD Cancer Research Fund [Y-MSDZD2021-0213] and National Ten-thousand Talent Program.

## Author contribution

H.Z. and X.Y.: conception/design; S.W., Y.W., C.Z., and K.L.: data analysis and interpretation and drafting of the manuscript. All authors participated in patient follow-up and contributed to data collection. All authors contributed to reviewing or revising the manuscript and approved the final version.

## Conflicts of interest disclosure

The authors declare no conflict of interest.

## Research registration unique identifying number (UIN)

NCT03892577.

## Guarantor

Haitao Zhao, Peking Union Medical College Hospital, No.1 Shuaifuyuan, Wangfujing, Dongcheng District, Beijing, 100730, People’s Republic of China. Tel.: +86 010 69152830. E-mail: zhaoht@pumch.cn.

## Data availability statement

All data supporting the results of the study can be found in the article and online supplementary material files. Further inquiries can be directed to the corresponding author.

## Provenance and peer review

Not commissioned, externally peer-reviewed.

## Supplementary Material
