# Supplementary material for: Conversion surgery intervention versus continued systemic therapy in patients with a response after PD-1/PD-L1 inhibitor-based combination therapy for initially unresectable biliary tract cancer: a retrospective cohort study
Source: Int J Surg. 2024 May 3;110(8):4608–16. doi: 10.1097/JS9.0000000000001540 (PMC11326034; doi:10.1097/JS9.0000000000001540)
Supplement: Supplementary file 2 [file js9-110-4608-s002.docx]

**Supplementary Table S1. Characteristics of two groups at the time when patients met the criteria for conversion surgery.**

| **Characteristic** |  | **No. (%)** |  | ***P* value** |
| --- | --- | --- | --- | --- |
|  | **Total**  **(N= 34)** | **Surgical group**  **(n=13)** | **Non-surgical group**  **(n=21)** |  |
| ﻿Median age (range) — yr | 61 (41-78) | 62 (41-78) | 59 (41-72) | 0.20 |
| ﻿Female | 13 (38.2) | 6 (46.2) | 7 (33.3) | 0.49 |
| ECOG PS |  |  |  |  |
| 0 | 23 (67.6) | 9 (69.2) | 14 (66.7) | ＞0.99 |
| 1 | 11 (32.4) | 4 (30.8) | 7 (33.3) |  |
| Child–Pugh grade |  |  |  |  |
| A | 33 (97.1) | 13 (100) | 20 (95.2) | ＞0.99 |
| B | 1 (2.9) | 0 | 1 (4.8) |  |
| Tumor response†  PR | 34 (100) | 13 (100) | 21 (100) | ＞0.99 |
| Clinical T stage‡  T1  T2  T3  T4 | 12 (35.3)  10 (29.4)  7 (20.6)  5 (14.7) | 3 (23.1)  5 (38.5)  3 (23.1)  2 (15.4) | 9 (42.9)  5 (23.8)  4 (19.0)  3 (14.3) | 0.65 |
| Clinical N stage‡  N0  N1 | 23 (67.6)  11 (32.4) | 9 (69.2)  4 (30.8) | 14 (66.7)  7 (33.3) | ＞0.99 |
| Clinical M stage‡  M0 | 34 (100) | 13 (100) | 21 (100) | ＞0.99 |
| cTNM stage‡  I  II  III  IV | 9 (26.5)  7 (20.6)  16 (47.0)  2 (5.9) | 2 (15.4)  3 (23.1)  6 (46.1)  2 (15.4) | 7 (33.3)  4 (19.1)  10 (47.6)  0 | 0.30 |
| ﻿CA19-9 (U/mL)  (median, IQR) | 21.1  (7.9-45.6) | 24.1  (5.8-43.4) | 20.4  (10.0-54.2) | 0.57 |

ECOG PS, Eastern Cooperative Oncology Group Performance Status; PR, partial response; CA19-9, Carbohydrate antigen 19-9; IQR, Interquartile range.

† Tumor response was evaluated per response evaluation criteria in solid tumors (RECIST) version 1.1.

‡ Clinical TNM staging (cTNM) was classified according to AJCC/UICC’s 8th edition TNM staging system.
