# Supplementary material for: Conversion surgery intervention versus continued systemic therapy in patients with a response after PD-1/PD-L1 inhibitor-based combination therapy for initially unresectable biliary tract cancer: a retrospective cohort study
Source: Int J Surg. 2024 May 3;110(8):4608–16. doi: 10.1097/JS9.0000000000001540 (PMC11326034; doi:10.1097/JS9.0000000000001540)
Supplement: Supplementary file 3 [file js9-110-4608-s003.docx]

**Supplementary Table S2.** Univariate and multivariate analysis of prognostic factors.

| Variable | Comparison | Overall survival | | |  | Progression-free survival | | |
| --- | --- | --- | --- | --- | --- | --- | --- | --- |
|  |  | Univariate | Multivariate | |  | Univariate | Multivariate | |
|  |  | *P* value | HR (95% CI) | *P* value |  | *P* value | HR (95% CI) | *P* value |
| Age (yr) | ≥ 60 vs < 60 | 0.20 |  |  |  | **0.01** | 0.47 (0.19-1.14) | 0.10 |
| Sex | Male vs. Female | 0.92 |  |  |  | 0.20 |  |  |
| ECOG PS | 1 vs. 0 | 0.30 |  |  |  | 0.84 |  |  |
| Child-Pugh grade | B vs. A | 0.90 |  |  |  | 0.74 |  |  |
| Extent of disease | Metastatic vs. Locally advanced | 0.15 |  |  |  | **0.09** | 1.51 (0.53-5.42) | 0.48 |
| Primary tumor site | GBC vs. ICC | 0.37 |  |  |  | 0.36 |  |  |
| CA19-9 level (U/mL) | ≥ 37 vs. < 37 | **0.09** | 1.85 (0.69-4.93) | **0.22** |  | 0.21 |  |  |
| Conversion surgery | Yes vs. No | **0.01** | 0.29 (0.10-0.90) | **0.03** |  | **0.0002** | 0.20 (0.06-0.55) | **0.003** |

Variables with *P* < 0.1 in the univariate analysis were incorporated into the multivariate analysis.

Abbreviations: ECOG PS, Eastern Cooperative Oncology Group Performance Status; CA 19-9, carbohydrate antigen 19-9; CI, confidence interval. ICC, intrahepatic cholangiocarcinoma; GBC, gallbladder cancer.
