# Supplementary material for: Conversion surgery intervention versus continued systemic therapy in patients with a response after PD-1/PD-L1 inhibitor-based combination therapy for initially unresectable biliary tract cancer: a retrospective cohort study
Source: Int J Surg. 2024 May 3;110(8):4608–16. doi: 10.1097/JS9.0000000000001540 (PMC11326034; doi:10.1097/JS9.0000000000001540)
Supplement: Supplementary file 4 [file js9-110-4608-s004.docx]

**Supplementary Table S3.** Therapeutic regimens, perioperative conditions and outcomes in surgical group.

| **Parameter** | **Patient no.** | | | | | | | | | | | | |
| --- | --- | --- | --- | --- | --- | --- | --- | --- | --- | --- | --- | --- | --- |
|  | **1** | **2** | **3** | **4** | **5** | **6** | **7** | **8** | **9** | **10** | **11** | **12** | **13** |
| Age (year) | 63 | 62 | 61 | 45 | 62 | 65 | 75 | 62 | 61 | 77 | 41 | 72 | 53 |
| Sex | Female | Female | Male | Female | Female | Male | Male | Female | Male | Male | Male | Female | Male |
| Diagnosis | ICC | ICC | GBC | ICC | GBC | GBC | GBC | GBC | ICC | GBC | ICC | GBC | ICC |
| cTNM ﻿stage‡ | T1bN0M1 (IV) | T1bN1M1  (IV) | T2aN1M1  (IVB) | T2N1M0  (IIIB) | T4N1M1  (IVB) | T3N1M1 (IVB) | T4N1M1 (IVB) | T3N1M1 (IVB) | T2N1M0 (IIIB) | T2bN2M1 (IVB) | T2N0M0 (II) | T4N1M1 (IVB) | T1bN1M1 (IV) |
| Unresectable reasons | Distant metastasis | Distant metastasis | Distant metastasis | Insufficient remnant  liver volume and multiple intrahepatic metastasis | Distant metastasis | Distant metastasis | Distant metastasis | Distant metastasis | Insufficient remnant  liver volume + multiple intrahepatic metastasis | Distant metastasis | Insufficient remnant  liver volume + multiple intrahepatic metastasis | Distant metastasis | Distant metastasis |
| PD-L1 expression† | 90 | 20 | 10 | 20 | 100 | 70 | 80 | 100 | 100 | NA§ | 0 | 80 | NA§ |
| ICI | Env | Cam | Cam | Sin | Tor | Tor | Tor | Tor | Pem | Tor | Dur | Sin | Dur |
| TKI | / | Len | Len | Len | Len | Len | Len | Len | Len | Len | / | Len | Len |
| Chemotherapy | Gemox | / | Gemox | / | Gemox | / | / | / | / | / | Gemox | / | / |
| Local therapy | TACE | TACE | / | TACE | RT | HAIC | / | RT | TACE; RT | RT | / | TACE | RT |
| Therapy line | 1 | 1 | 2 | 1 | 1 | 2 | 1 | 1 | 1 | 1 | 2 | 1 | 1 |
| Interval from initial ICI to surgery (Months) | 8.9 | 7.7 | 6.7 | 5.9 | 13.7 | 5.3 | 9.5 | 11.6 | 4.4 | 2.9 | 7.4 | 1.8 | 5.8 |
| Interval from TKI discontinued to surgery (days) | 16 | 15 | 12 | 10 | 9 | 11 | 12 | 21 | 16 | 7 | / | 11 | 7 |
| Interval from ICI discontinued to  surgery (days) | 31 | 29 | 22 | 19 | 23 | 21 | 26 | 29 | 21 | 8 | 27 | 22 | 28 |
| Downsizing  (RECIST) | PR | PR | PR | PR | PR | PR | PR | PR | PR | PR | PR | PR | PR |
| Pre-operative cTNM stage ‡ | T1aN0M0 (IA) | T1aN0M0 (IA) | T2N0M0 (IIA) | T2N1M0  (IIIB) | T4N0M0  (IVA) | T3N0M0 (IIIA) | T4N0M0(IVA) | T3N0M0 (IIIA) | T2N1M0 (IIIB) | T2bN0M0 (IIB) | T2N0M0 (II) | T3N1M0 (IIIB) | T1aN1M0 (IIIB) |
| Resection extent | S4 resection | Cholecystectomy+ S4b resection + abdominal wall metastasectomy | Cholecystectomy + segment 4b,5 resection Hilar lymphadenectomy | Left trisectionectomy + Hilar lymphadenectomy | Cholecystectomy+ segment 4b,5 + Hilar lymphadenectomy | Cholecystectomy + S4b,5 resection + Hilar lymphadenectomy | Cholecystectomy + S4b,5 resection + Hilar lymphadenectomy | Cholecystectomy + segment 4b,5 resection + Hilar lymphadenectomy | Cholecystectomy + left hemihepatectomy + portal vein thrombectomy + Hilar lymphadenectomy | Cholecystectomy+ Hilar lymphadenectomy | Left lateral sectionectomy + two intrahepatic metastasectomy | Cholecystectomy + S4b,5 resection + Hilar lymphadenectomy | Cholecystectomy + left hemihepatectomy + Hilar lymphadenectomy |
| Surgical approaches | Laparoscopic | Laparoscopic | Laparoscopic | Laparotomy | Laparoscopic | Laparotomy | Laparotomy | Laparotomy * | Laparotomy | Laparoscopic | Laparoscopic | Laparotomy | Laparoscopic |
| Operation  time (min) | 240 | 245 | 205 | 280 | 90 | 220 | 265 | 190 | 225 | 40 | 225 | 220 | 170 |
| Blood  loss(ml) | 200 | 200 | 100 | 200 | 130 | 200 | 200 | 100 | 200 | 30 | 200 | 130 | 100 |
| Postoperative complications  ≥ Clavien-Dindo† 3a﻿ | / | / | / | / | / | / | / | / | / | / | / | DVT+  anaphylactic shock | / |
| Postoperative hospital stay (days) | 5 | 6 | 10 | 8 | 3 | 8 | 10 | 30 | 8 | 8 | 6 | 24 | 7 |
| Postoperative systemic therapy | Env + Gemox | Cam + Len | Cam + Len + Gemox | Sin + Len | Tor +Len | Tor +Len | Tor +Len | Tor +Len | Pem+ Len | Tor +Len | Dur + Gemox | Sin + Len | Dur + Len |
| Resection margin | R0 | R1 | R0 | R0 | R0 | R0 | R0 | R0 | R0 | R0 | R0 | R0 | R0 |
| pTNM‡ | T1aN0M0  (IA) | T1aN0M1  (IV) | pCR | T2N0M0  (II) | T2bN0M0  (IIB) | T2bN0M0  (IIB) | T3N0M0  (IIIA) | T2bN0M0 (IIB) | T2N0M0  (II) | pCR | T2N0M0 (II) | T3N1M0 (IIIB) | T0N1M0  (IIIB) |
| PFS  length  status | 56.0  No PD | 54.7  No PD | 17.7  PD | 38.7  No PD | 39.7  No PD | 19.6  PD | 18.4  PD | 37.6  No PD | 10.5  PD | 24.6  No PD | 16.3  PD | 20.0  No PD | 17.6  No PD |
| Recurrence site | / | / | Hepatic hilar lymph node | / | / | Colon | Liver | / | / | / | Liver | / | / |
| OS  length  status | 60  Alive | 57.5  Alive | 35.4  Died | 42.7  Alive | 45.9  Alive | 34.6  Died | 19.8  Died | 40.5  Alive | 10.5  Died | 28.8  Alive | 29.2  Alive | 22.8  Alive | 18.0  Alive |
| Death during follow-up  tumor-related  non-tumor related | / | / | Yes¶ | / | / | Yes | Yes | / | Yes¶ | / | / | / | / |

ICC, intrahepatic cholangiocarcinoma**;** GBC, gallbladder cancer; TKI, tyrosine kinase inhibitor; ICI, immune checkpoint inhibitors; RT, radiotherapy; Len, lenvatinib; Envolizumab, Env; Sin, sintilimab; Cam, camrelizumab; Tor, toripalimab; Pem, pembrolizumab; Dur, Durvalumab; TACE, transarterial chemotherapy and embolization; HAIC, hepatic artery infusion chemotherapy; RT, radiotherapy; Gemox, Gemcitabine plus Oxaliplatin; PR, partial response; PD, progressive disease; pCR, pathologic complete response; PFS, progression-free survival; OS, overall survival; RECIST, response evaluation criteria in solid tumors; DVT, deep venous thrombosis.

† PD-L1 expression was evaluated by combined positive score (CPS).

§ NA, unknown and not tested.

‡ According to AJCC/UICC’s 8th edition TNM staging system, clinical staging (cTNM), pre-operative clinical staging (cTNM) and pathological staging (pTNM) were classified.

* Laparoscopic exploration and then was converted to laparotomy.

¶ Alimentary tract hemorrhage.
